# Aconite in Pneumonia

**Published:** 1879-11-20

**Authors:** A. C. F. Rabagliati


### *ACONITE IN PNEUMONIA.*

BY A. C. F. RABAGLIATI, M. A., M. D.

The cases of pneumonia cut short by the use of tincture of aconite which Dr. Dobie has lately recorded in the Practitioner appear to me deserving of some remarks, which I shall attempt to keep within as short a compass as possible. I have no doubt at all the cases were really pneumonias, and the treatment is such as is not only recommended in some text-books of therapeutics, but I should say is in pretty general use among medical men, though not yet perhaps so much so as it ought to be. It has been my practice for years to treat incipient pneumonias and also other simple inflammations by aconite, which however I have found it convenient to use of one-tenth of the strength of the pharmacopœial tincture, the remaining nine-tenths being rectified spirit. I think if Dr. Dobie had used this preparation he would not have induced the vomiting which he describes in cases III. and IV., and which is no essential part of the cure.

I should like to say a few words on the rationale of the use of aconite, and shall do so in language similar to what I used in addressing to your readers some remarks on the question, "Are there Laws of Therapeutics?" In my view aconite follows the universal law of agents (not merely therapeutical remedies) competent to affect the economy, viz., that it acts by action and reaction, which are contrary to one another. The action of aconite is the same as that of cold; it depresses the vital power of the sympathetic nerves, and contracts the vaso-motor muscular fibres. The reaction of aconite (or, properly speaking, of the economy affected by aconite) is shown in relation of the spasm, and in congestion of the capillary vessels. If one attends only to the latter occurrence, one may say, aconite causes the phenomena of the (simple) feverish state. In point of fact, however, this is only half the truth, which is stated fully thus: aconite causes first spanæmia, and second, congestion of the capillary vessels. Now what is (simple) inflammation? Is it not the reaction due to exposing the body to cold? And are not the steps of the process first spanæmia of the exposed part, and second congestion of the same? The latter condition we are in the habit of calling inflammation, though only by overlooking the fact that it is secondary, and not a primary occurrence—an error, by the way, that Cullen did not fall into. Aconite, then, cures inflammation because its action depresses the vaso-motor system, and therefore lowers the excitement of the febrile reaction. For which reason the dose should be small, so small as not to induce any secondary wave of reaction, which might leave the patient as bad as he was before. Being small, the dose should be frequently repeated; and I am in the habit of administering it, not every hour or half hour, but every ten or fifteen minutes, till lowering of pulse and temperature, moisture of skin, and sleep, are induced. Not once, but many times, have I been able thus to check incipient pneumonia, peritonitis, pleurisy, and tonsilitis; and in children particularly the effects are marvelous. The induction of sleep is difficult to explain. I do not think it is a direct effect of aconite, but rather an indirect one, obtained by combating the excitement, and allowing rather than inducing nature to complete the cure by rest. Sweating seems also to be an indirect effect of the administration, to be explained in some similar way.

Next comes the question, Why does aconite act in pneumonia on the capillaries of the lung, in pleurisy of the pleura, in tonsilitis of the tonsil, etc.? Because, it is replied, these parts when inflamed are more susceptible than when they are in their normal state, just as they are more susceptible to painful pressure. Aconite has a wide range of action, but it is determined to act on a given part of the capillary system by the susceptibility of that part.

Now, to show further that this view of action and reaction is sound, let me remind you that the reaction caused by cold can be itself cured by the action of cold. Judicious "packing" will resolve a pneumonia about as well as aconite. Again: excessive pressure will cause inflammation of the skin as its reactionary effect, and I have, on one occasion, which I shall always remember, cured the inflammatory erysipelas which followed excision of the knee-joint in a young woman by a carefully applied bandage. The water used for packing in the former case should not be too cold, neither should the bandage in the latter be too tightly applied; otherwise reaction might in turn be set up and the inflammatory symptoms increased. Supposing the erysipelas in the case just referred to to have been due to over-pressure, these instances of treatment are not covered by the formula *similia similibus*, but must be stated as *eadem iisdem* or *identical by identical.* Even then, however, the formula would be but an empirical one, which is explained when action and reaction, its simpler expression, are considered and understood. Neither is it true to say, as some do, "a moderate dose acts as you describe, but a large one has only what you call a secondary action." For, first, what is a large dose, and what a small, and what a moderate one? And, second, when the dose is big enough, only the primary action is induced, since the patient does not live to have the secondary; just as a man exposed all night in the snow has neither inflammation nor frost bite, since he is killed before he has time to have either. Supposing one were to argue from that, that cold has an opposite action in small quantities to what it exerts in large quantities! Thirdly, just as the mass of medical men to-day think of inflammation as a congestion simply overlooking the spanæmia which precedes that condition (and how many of us have given due attention to the subnormal pulse and temperature which succeed the congestion?) so, I am convinced, have many others overlooked the depression induced by aconite previously to the induction of the feverish state, and hence have said, "Aconite induces the feverish state." There is reason for thinking that the reaction of a remedy or agent is proportional to the action of the same; but I will not weary your readers by repeating at length what has been said on a former occasion. Meantime I think I have shown that—

1. Aconite, like other agents, has on the economy an action and a reaction. The former is a spanæmic, the latter a congestive action.

2. Small and large doses have only an apparent, not a real contrariety of action.

3. Aconite acts in simple inflammations by combating the reaction of cold, which is commonly, but only by oversight, called inflammation.—*The Practitioner.*

